# The provision of the baby box was associated with safe sleep practices in a low-resource community: a randomized control trial in Ecuador

**DOI:** 10.1186/s12887-022-03832-y

**Published:** 2023-01-19

**Authors:** Hartley Feld, Janeth Ceballos Osorio, Marisol Bahamonde, Thomas Young, Pablo Boada, Mary Kay Rayens

**Affiliations:** 1 College of Nursing, University of Kentucky, 751 Rose Street, KY 40536-0232 Lexington, USA; 2 Department of Pediatrics, College of Medicine, University of Kentucky, Lexington, KY, USA; 3 Universidad San Francisco de Quito, Quito, Ecuador; 4 Fundación Hombro a Hombro, Quito, Ecuador

**Keywords:** Infant sleep environment, Baby box, SUID prevention, Ecuador

## Abstract

**Background:**

Sudden Unexpected Infant Deaths (SUID) can occur between 1 month and 1 year of age and are inequitably distributed with a greater burden in populations with numerous health disparities. Modifying the infant sleep environment to promote safe sleep is the most effective risk reduction strategy to reduce SUID. The provision of baby boxes with a mattress and infant supplies has been part of a larger anti-poverty social justice maternity package for decades in Finland. While infant mortality rates have generally improved after the maternity package was introduced, little is known about whether the provision of the baby box increased safe sleep practices. The purpose of the study was to evaluate whether the provision of a Finnish-style baby box reinforced safe infant sleep practice in the home in a low-resource community in Ecuador.

**Methods:**

In this longitudinal randomized controlled trial all participants received the same safe sleep education in their third trimester of pregnancy (*n* = 100). This was followed by randomization into two groups; the control received a diaper bag and newborn gifts, and the intervention group received a baby box and the same gifts at each timepoint. Four infant sleep practices (room sharing, bed sharing/co-sleeping, position, and soft items in the sleep environment) were assessed at 1 month and 1 months post-delivery during a home visit where safe sleep education was also reinforced with both groups.

**Results:**

Those in the baby box group were 2.5 times more likely to report safe sleep practices compared with mothers in the diaper bag group at 1 month (odds ratio [*OR*] = 2.45 and 95% confidence interval [*CI*]: 1.03–5.86; χ^2^ = 4.1, *p* = .043). The group difference was also present at 6-months post-birth: those in the baby box group were 2.9 times more likely to report safe sleep practices compared with those in the diaper bag group (*OR* = 2.86 and 95% *CI*: 1.16–7.05; χ^2^ = 5.2, *p* = .022).

**Conclusions:**

While not all participants used the box regularly, the mothers who received the box were more likely to practice safe sleep at 1 month and 6 months. This suggests the baby box may have served as an important prompt towards safer infant sleep practice.

**Trial registration:**

(Clinical Trial Registry, per clinicaltrials.gov: not applicable under 42 CFR 11.22(b) as the study Facility Location was not in the United States (took place in Ecuador), does not involve FDA IND or IDE, and does not involve a drug, biological or device product that is manufactured in and exported from the US for study in another country. The University of San Francisco Quito, Research Ethics Committee in Human Beings approved the study, #2017- 127 M. The University of Kentucky Office of Research Integrity also approved the study, IRB # 42965).

**Supplementary Information:**

The online version contains supplementary material available at 10.1186/s12887-022-03832-y.

## Introduction/background

Sudden Unexpected Infant Deaths (SUIDs) that are sleep-environment related are diagnosed as suffocation, entrapment, or unknown etiology [1]. Those where the causes of death are investigated and remain of unknown etiology, make up the majority of SUIDs and are categorized as sudden infant death syndrome (SIDS) and 90% of these deaths occur prior to 6 months of age [1]. In high income countries SUIDs tend to be inequitably distributed, where the burden is greater in populations with numerous health disparities; specifically those with socioeconomic, racial/ethnic, and educational disparities are most at risk of SUIDs [2].

In the late 1980s and early 1990s researchers in several European countries and New Zealand found many SUIDs were linked to the prone sleep position, which was followed by mass public health campaigns promoting “Back to Sleep”, whereby parents and care givers were instructed to place their infants in a supine or face up position to sleep [3–5]. These strategies led to a dramatic reduction in infant deaths and increased scrutiny of the sleep environment in other countries. Supine sleeping was later widely promoted globally and adopted in the United States (US) as were other sleep environment recommendations to reduce SUID, but less is known about SUID rates and safe infant sleep practices in other parts of the Americas [4, 5].

In 2002 investigators assessed sleep position recommendations medical professionals gave to new parents in 16 countries in Latin America and the Caribbean and found that only 25.7% recommended supine sleep position in the home [6]. More recently researchers in Argentina reported 7% of all infant deaths younger than 1 year were attributed to SUIDs, and in 2010 almost 60% of Argentinian infants slept in the supine position [7, 8]. Other studies in South America indicate that far fewer slept in the supine position; only 24% in Brazil, and 36% in Peru reported placing infants on their back to sleep [9, 10]. Bed-sharing or co-sleeping in the family bed was widely practiced in Peru (75%) and less so in Brazil (48%) [9, 11].

The current study took place in Ecuador where infant sleep practice was an understudied area. Although SUIDs were collected in each province annually by the Ministerio de Salud Publica (MSP), they were not commonly published or reported. One of our co-authors, Dr. Bahamonde, requested raw mortality data (unpublished) from the MSP for all provinces that reported data in 2018. In Santo Domingo de los Tsa’chilas province, where the study took place, the highest number of SUIDS deaths was reported (4 in a population of 442,788 people). In the absence of published data, anecdotal information was also collected from clinical staff where the study took place, a small clinic in a peri-urban and low-resource community. They indicated that in their patient population there were 4 infant deaths in the last 5 years, 3 of which were attributed to the sleep environment.

The Ecuadorian MSP published practice recommendations to reduce sleep-related deaths between 1 month and 1 year of age and cite the American Academy of Pediatrics (AAP) 2016 recommendations [12, 13]. In addition to the supine position, modifying other aspects of the infant sleep environment was a critical feature of the 2016 AAP recommendations. These included the use of a firm sleep surface, room-sharing without bed-sharing, and the avoidance of soft bedding and overheating [14]. AAP safe sleep education directed towards the primary caregiver is the most common intervention to reduce SUIDs in the US and is also the recommended pediatric practice in Ecuador to be performed during the first well-child infant visit 3 to 5 days after birth and every visit in the first year of life, with a greater emphasis in the first 6 months [12, 13].

Published accounts of prenatal safe infant sleep education in the US demonstrated an increase in knowledge and intended adherence particularly when receiving advice from a healthcare provider, but often does not translate into sustained safe sleep practice after the infant is born [15, 16]. Researchers suggest that reinforcing caregiver knowledge and addressing barriers to safe sleep after the infant is born may assist families to maintain safe sleep practice over time [16]. More research is needed to prevent sleep related infant deaths in many contexts and to better understand interventions that promote behavior change and the uptake of sustained safe sleep practices. Key characteristics of effective interventions to change caregiver behaviors and improve the sleep environment have been identified using Grol’s Conceptual Framework as model to guide safe infant sleep interventions [17]. His work focuses on identifying and removing barriers, as well providing incentives to promote behavior change. The intervention needs to be innovative, attractive, accessible, and consider all levels of influence such as the social context, the education of the professionals delivering the intervention, and existing barriers for the caregiver [17]. These factors may be even more critical when implementing an intervention in a cross-cultural context, especially when sleep guidelines and recommended practices are developed in high income countries and implemented in low- or middle-income countries.

One intervention strategy used in numerous countries to mitigate risks in the sleep environment for low-income families was by promoting a portable, culturally acceptable, and affordable sleep device paired with comprehensive maternal and child health education and care. New Zealand’s indigenous Māori community had high rates of infant mortality and SUID prior to 2005, which was largely attributable to smoking and co-sleeping [18]. When smoking cessation efforts largely failed, the Māori worked with health professionals to create a culturally resonant alternative to reduce harm. They crafted the wahakura flax basket to allow for a little separation and a safer shared sleeping environment (as assessed at 1 month), a larger community engaged approach to health promotion, and over time a 29% reduction in infant mortality [18]. The wahakura basket was difficult to mass produce and a plastic version called the Pēpi-PodⓇ emerged as a safe sleep enabling device used to co-sleep in New Zealand and Australia [19].

In Finland another strategy to reduce social inequality and improve maternal and child health outcomes for people in poverty included the provision of a cardboard baby box as a portable sleep space as one part of a much larger investment in prenatal care, education and social programs in the 1930s [20]. These are known as Finnish baby boxes and are decorated with a baby-friendly design, come with a mattress and sheet as well as other essential newborn items, they refer to as the “maternal package”. The box passed Finnish safety standards and was considered a safe sleep environment that parents were encouraged to use as their first crib, however there were no specific safe sleep guidelines or education included [21]. In the 1940s the Finnish government made the provision of the free maternal package conditional, in that pregnant people could only participate if they attended antenatal care before the end of the 4th month of pregnancy [22]. After the national investment in many programs to reduce social inequality, one of which was the maternal package, Finland’s infant mortality rates dropped to one of the lowest globally [23]. In 2020 Finnish researchers Koivu et al. published an extensive history of the baby box, covering global distribution, adaptations, as well as their promising potential to reduce poverty-related stigma [22]. Her team also refers to the cultural significance of their baby box programs as an embodiment of “Finland’s commitment to public policies that value mothers, babies and families”(p. 78) [22].

It is worth noting that other northern European countries also reduced infant mortality rates substantially at the same time as Finland and did not adopt the baby box but did invest in social programming and healthcare [23]. The provision of the Finnish-style baby box without the larger investment in social care was later adopted by over 60 countries with the hopes of reducing infant mortality rates [22]. There is a lack of clear evidence that the provision of the Finnish-style baby box leads to safe sleep practices, reduces SUIDs, or reduces infant mortality, thus a more intentional examination of the provision of the box in countries where they are not in use is warranted [24].

Many states in the US have been providing a similar Finnish-style box and infant supplies, herein just referred to as baby box, to incentivize and engage low-income families to attend prenatal care [25]. The American Academy of Pediatrics (AAP) did not endorse the baby box as a safe sleep device yet the provision of the box has been promoted as a pragmatic and affordable solution, especially for people who lack of an identified place for the infant to sleep, as this has been implicated as a contributing factor to co-sleeping [26].

Research surrounding the efficacy of the baby box to promote safe sleep has been limited. In one study in the US researchers found that the provision of the baby box in combination with personalized face to face sleep education by a nurse reduced the rate of bed-sharing by 25% in the first week of life, as compared to standard discharge written post-partum instructions [27]. Since the intervention in this study included different delivery methods and intensity of safe sleep education than the control group, it is difficult to determine if the provision of the baby box made a difference [27]. These researchers were the first to find that 59% of breastfeeding mothers reported that the box made breastfeeding easier, but again this study was limited to only the first 8 days of life so we don’t know if that was sustained [27]. Other researchers have questioned whether the box inhibits breast feeding as compared to those who co-sleep or have other sleep devices [28]. While these findings are promising, they do not elucidate whether the provision of the box alone was a key factor to adopting a safer sleep practice or if the intervention was sustained beyond the first week of life. Other issues regarding the safety of the box have not been well studied. There are concerns about the integrity of the cardboard, the placement of the box, and ventilation and air quality may also be of concern because the height and impermeability of the box as compared to cribs and bassinets with mesh siding [23, 28].

An extensive review of the literature revealed no studies to our knowledge evaluating the provision of baby boxes to promote safe sleep in randomized controlled trial with systematic follow up in the home at several time points. Finnish researchers have interviewed numerous organizations that distribute baby boxes globally and found a governmental program in Chile has distributed 140,000 baby boxes over a 9-year period and another program reported operating in several Latin American countries [22]. Many of these programs in middle and low-income countries use the boxes and newborn items as part of a larger umbrella of social protection, whereby the boxes are provided in-kind for those most vulnerable to support parents and their newborns [29]. While many use them conditionally, similar to Finland, to incentivize antenatal care or to be administered if they receive education as a prompt for other behavior change, to improve brain development, parenting, breast feeding or safe sleep, but few measure the impact of their intervention systematically [22].

### Purpose

The present study fills an important gap in pediatric literature as it evaluates whether the provision of the baby box offers any additional benefit over that of safe sleep education alone. To assess this we provided the same AAP safe sleep education to all participants, and then randomized them to receive either a diaper bag or the baby box. The purpose of this study was to promote safe infant sleep practices and to assess specifically if the provision of the baby box improved safe sleep practices in a low-resource peri-urban community in Ecuador. The aims were: 1) to describe the participants’ intentions regarding the infant sleep environment prior to the AAP education including location, use of soft items, position, and risk or protective factors such as maternal cigarette, alcohol and other drug use and breastfeeding; and 2) to evaluate the changes over time and between intervention groups in prenatal intentions and post-birth behaviors related to infant sleep practices at 1 month and 6 months. Our hypothesis was that the provision of the baby box would decrease maternal-infant bed-sharing and improve sleep safety by deterring the use of loose or heavy blankets or soft items in the place where the infant slept. We did not anticipate that there would be a difference in the sleep position of the infant based on the provision of the box.

## Methods

### Setting

This study took place at a community health center in a low-resource community in Santo Domingo de los Tsa’chilas, Ecuador from 2018 to 2020. In 2007 the University of Kentucky partnered with this health center to supplement primary care and promote community health. The center is privately supported by a non-governmental organization (NGO) and provides low cost or free primary health care, including care during pregnancy. This study originated in part due to concerns from the Ecuadorian health center clinic staff, who indicated unsafe infant sleep practices are common in the community, with several of their patients reporting infant deaths related to the sleep environment. The baby boxes were donated by a US company to the NGO and were not modified for use in this study, research funds were used to purchase the diaper bags and newborn gifts. This region has a similar climate year-round so the use of blankets or items in the sleep environment was not influenced seasonally. Additionally, this region is very humid so safety and integrity of the box in this context was a priority of the research team. Participants were told to discontinue use if the box was not fully intact, and the box was assessed by the study personnel at each time point.

### Design and sample

There are no previous studies in this community regarding safe infant sleep practices. Data from studies in other South American communities with similar sociodemographic characteristics were used to determine sample size of 50 in each group using two outcome measures, bed-sharing and position.

#### Bed-sharing

The proportion of bed-sharing in a Southern community in Brazil was 48.3% [11]. The study from Temple university which included a face-to-face education on safe infant sleeping practices and the provision of the baby box reduced the rate of bed-sharing by 25% overall, and by 50% among breast-feeding mothers [30]. Assuming the same proportion of bed-sharing in our community, to detect a difference of 25% in bed-sharing between the group receiving the baby box and the control group, with 80% power using 5% level of significance, two groups of 45 participants would be needed [31].

#### Infant sleep position

A randomized clinical trial of a single educational intervention at hospital discharge on infant position to sleep in the maternity ward in Porto Alegre, Brazil showed a proportion of 24% of infants placed back to sleep in the control group and 42.9% in the intervention group [10]. A prospective study in Argentina of back to sleep education found a change from 49 to 90% of infants placed back to sleep over 10 years [32]. Assuming a proportion of infants placed prone to sleep of 76%, to detect a difference of 25% on infant sleep position between the group receiving the baby box and the control group, with 80% power using 5% level of significance, two groups of 45 participants would be needed [31].

#### Drop-out-rate

After discussion with clinical staff at STSG health center we estimated a dropout rate of 10%. We increased our numbers by 10% adding 5 participants per group, two groups of 50.

The design of this randomized intervention study was longitudinal, with assessments in the third trimester of pregnancy and again at 1 and 6 months after birth. The 6-month designation was based on the MSP guidelines to reinforce safe sleep each visit in the first year of life, with emphasis on the first 6 months [13]. The randomization was used to determine whether the participant received a baby box or diaper bag, and there was stratification within the randomization schedule so that each group would have the same percentage of primiparous mothers. Participants were recruited from the health center and after informed consent was given, they were asked to complete the pre-intervention prenatal survey by the nursing staff. In addition to demographic and personal variables, the survey included multiple items to assess the prenatal intention related to sleep location, position, and items in the place where the infant slept. Inclusion criteria were that the participant had to be an established patient of the health center, in their third trimester of pregnancy, and sixteen years or older. Participants were excluded if they did not agree to a follow-up home visit. After the pre-intervention survey participants received an educational intervention, which consisted of the 9-minute video from LivingLegacyPro entitled “Safe Sleeping for your Infant” [33], followed by face-to-face education using the Healthy Children handout entitled “How to Keep your Sleeping Baby Safe: AAP Policy Explained” [34]. The participant also received a Safe Sleep Environment handout from the NIH Safe to Sleep campaign [35]. All education and materials were in Spanish and delivered by health center Ecuadorian clinical staff, who were trained by the Ecuadorian pediatrician on the research team. Investigators and clinical staff involved in the study all completed the collaborative institutional training initiative (CITI) on human subjects research and were blinded during the prenatal educational intervention, as the randomization occurred after the education. Additionally, the research team did not directly participate in any aspects of the education or follow up with participants.

**Fig. 1:**
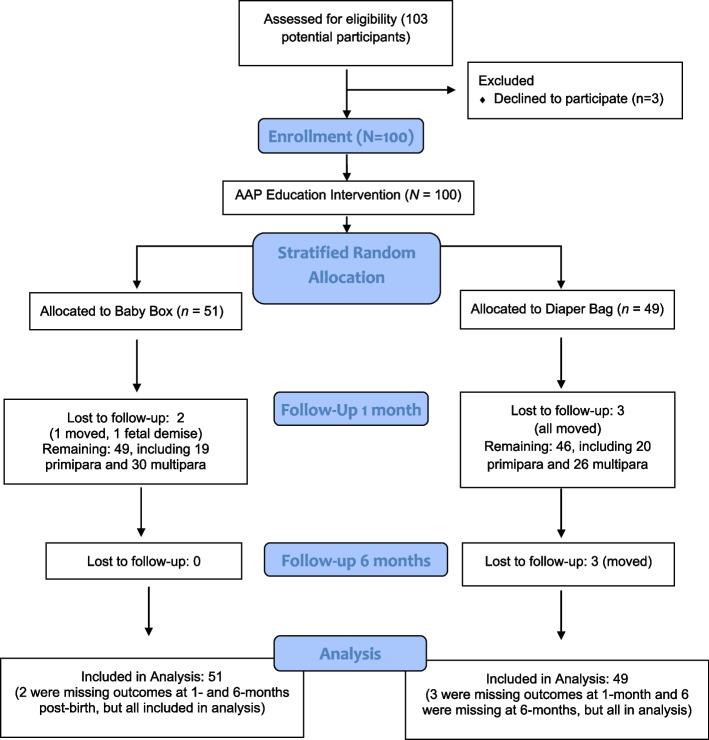
CONSORT diagram for baby box and AAP safe sleep education intervention study

While 103 eligible pregnant patients were invited to participate in the study, 3 declined (see Fig. 1 for CONSORT diagram). After completing the education portion the participants were randomized to one of two groups, with the goal of 50 participants in each. The case group participants received a baby box with newborn care supplies and instructions for how to use it as an infant sleep surface; those randomized to the control group received a diaper bag with the same newborn care supplies. Allocation was concealed and performed by opening a sealed envelope in numbered sequence. As parity may have influenced the primary outcomes, the randomization was done separately within two strata: participants who were primiparous versus those who were multiparous. The items received by both groups had similar monetary value and were described as gifts for participation in the study to reduce the possibility of a placebo effect.

Nursing staff tracked all participants due dates and contacted them to arrange for a home visit at 1 month and 6 months of age. A trained member of the clinical staff went to the home at each interval and completed a post-intervention survey to assess participant behaviors and assessed the condition of the box to assure safety. At the end of the visit the clinical staff reviewed and reinforced the AAP infant safe sleeping recommendations and answered any questions posed by the participants. During the one-month visit participants were provided a Safe Sleep card from the NIH Safe to Sleep campaign [36]. At the end of the six-month visit all participants received a small gift for the baby in acknowledgement for participation in the study.

### Measures

Demographic factors included in the baseline survey administered in the third trimester of pregnancy included maternal age, race/ethnicity (which was dichotomized to ‘mixed/Mestizo’ vs. ‘other race/ethnicity^1^’ to capture the predominant racial/ethnic composition of the area), family income (a binary choice between less than or equal to minimum monthly wage vs. greater than minimum), marital status (dichotomized as married/partnered vs. other), parity (primiparous vs. multiparous). The baseline survey also included the 2-item Patient Health Questionnaire (PHQ-2) Scale, as a brief tool for depression screening. The validity of this instrument has been assessed previously [37]. In the present study we used the suggested cut-off score of 3 or greater as an indicator of possible depressive symptoms. Finally, the baseline prenatal survey also included items to assess breastfeeding intention and maternal use of cigarettes, alcohol, and other drugs. The Survey of Well-being of Young Children (SWYC) was used to screen for smoking and alcohol/drugs, asked via two questions: ‘Do you smoke?’ and ‘In the last year, have you ever drunk alcohol or used drugs more than you meant to?’ both with a yes/no response option [38]. These two items were combined into a single item indicating maternal use of cigarettes, alcohol, or other drugs.

The items used to assess sleep prenatal intentions and post-birth reported behaviors are shown in Table 1. The items included in the instrument were the infants sleeping place (with distinction between sleeping in the bed with their mother, or in a separate place), sleep position, and items included (or not) in place where the infant slept. Although the exact wording of the items changed between the prenatal and post-birth period (to shift the focus from *intention* during pregnancy to *behavior* during the postpartum period) the purpose of each item in the instrument was to evaluate whether the typical infant sleeping place was in a separate location compared with the parent(s), with the infant on his/her back, and without loose/heavy blankets or pillows or stuffed animals in sleeping place. One point was added for each best practices response, and total scores of either 2 or 3 were considered at each time point to represent a safer sleeping option (compared with 0 or 1). This cutoff was chosen since there were relatively few scores that attained the maximum safety score of 3, regardless of group or timepoint.

**Table 1:** Scoring for safe sleep items during the prenatal and post-birth periods

Item	Prenatal (Intention)	Post-Birth (Reported Behaviors)
Sleeping Place	1 point for ‘*bassinet*,’ ‘*crib*,’ or ‘*other*’ place; 0 for ‘*mother’s bed*’	1 point if ‘*bassinet*,’ ‘*crib*,’ or ‘*other*’ place were ‘*always*’ or ‘*very often*’ used; 0 if ‘*mother’s bed*’ was ‘*always*’ or ‘*very often*’ used
Sleeping Position	1 point for ‘*back*;’ 0 for ‘*belly*’ or ‘*side*’	1 point if ‘*back*’ was ‘*always*’ or ‘*very often*’ used; 0 if ‘*belly*’ or ‘*side*’ was ‘*always*’ or ‘*very often*’ used
Items in Sleeping Place	1 point for ‘*no*’ to pillows, stuffed animals, or loose/heavy blankets in sleeping place; 0 for a ‘*yes*’ response to this	1 point for pillows, stuffed animals, or loose/heavy blankets used ‘*rarely*’ or ‘*never*;’ otherwise 0 for this
*Total Score at Each Survey (sum of the three items)*	*Range 0–3*^a^	*Range 0–3*^a^

^a^higher total scores indicate safer sleeping intentions or behaviors

### Data analysis

The study variables were summarized using means and standard deviations or frequency distributions, as appropriate. Group comparisons of maternal demographic or personal factors and prenatal sleeping intentions were done using the two-sample t-test or chi-square test of association. For a few comparisons with small expected cell counts, Fisher’s exact test was used as an alternative to the chi-square test of association. To evaluate changes in safer sleeping practices over time and between group differences, this binary outcome that was differentiated between safer (i.e. scores of 2–3 on the sum of the indicators for the three items) and less safe (i.e. scores of 0–1) sleep intentions or practices was assessed for the main effects of time and group and their interaction using generalized estimating equation (GEE) modeling. This type of modeling is an extension of logistic regression for longitudinal designs. Covariates included in the GEE model were age, race/ethnicity, family income, marital status, parity, PHQ-2 indicator, and the indicator for maternal use of cigarettes, alcohol, or other drugs. Post-hoc analysis was conducted using pre-specified contrasts. We were interested in differences between the two groups at each assessment rather than differences within a given group over time. We *pre-specified* the goal to make group comparisons at each timepoint should the significance of the interaction term warrant this (or to make the group comparison averaged across all three timepoints if only the main effect of group were significant in the model). With this strategy, we were able to limit the overall Type I error by minimizing the number of pairwise comparisons evaluated via contrasts. Data analysis was conducted using SAS, v. 9.4 with an alpha level of .05 for inferential testing.

## Results

The average age of all participants was 24.2 years (SD = 6.2), the range of ages was from 15 to 41; see Table 2. Most participants were of a mixed/Mestizo ethnicity (*n* = 82; 82%) and had a family income that was at most equal to the minimum wage (*n* = 89; 89%). The majority were married/partnered (*n* = 81; 82%) and were pregnant with a second or later child (*n* = 65; 65%). Nearly one-fourth had a PHQ-2 depression score that met or exceeded the cut-off of 3 (*n* = 22: 22%). All participants planned to breastfeed their infant and relatively few indicated use of cigarettes, alcohol, or other drugs (*n* = 7; 7%).

**Table 2:** Participant demographics and prenatal intentions, with intervention group comparisons (*N* = 100)

Demographics	Full Sample Mean (SD) *or* n (%)	Intervention Group		Test statistic (***p*** value)
		*Baby Box* *(n = 51)*	*Diaper Bag* *(n = 49)*	
Maternal age (years)	24.2 (6.2)	24.6 (6.4)	23.8 (6.1)	|t| = 0.6 (.55)
Mixed/Mestizo Ethnicity	81 (81.8%)	47 (92.2%)	34 (70.8%)	χ^2^ = 7.6 (.006)
Family Income Less Than or Equal to Minimum Wage	89 (89.0%)	45 (88.2%)	44 (89.8%)	χ^2^ = 0.1 (.80)
Married or Partnered	81 (81.8%)	45 (88.2%)	36 (75.0%)	χ^2^ = 2.9 (.088)
Multiparous	65 (65.0%)	33 (64.7%)	32 (65.3%)	χ^2^ < 0.1 (.95)
High PHQ-2 Score	22 (22.2%)	13 (25.5%)	9 (18.8%)	χ^2^ = 0.6 (.42)
Intention to breastfeed	100 (100.0%)	51 (100.0%)	49 (100.0%)	–
Maternal Cigarette, Alcohol, Other Drug Use	7 (7.0%)	2 (3.9%)	5 (10.2%)	* (.26)
**Safe Sleep Intentions during Third Trimester**				
Place where infant will sleep				
Mother’s Bed	75 (75.8%)	36 (72.0%)	39 (79.6%)	* (.33)
Bassinet	3 (3.0%)	3 (6.0%)	0 (0.0%)	
Crib	20 (20.2%)	10 (20.0%)	10 (20.4%)	
Other	1 (1.0%)	1 (2.0%)	0 (0.0%)	
Sleeping Position				
Belly	20 (20.2%)	8 (15.7%)	12 (25.0%)	χ^2^ = 5.3 (.070)
Side	51 (51.5%)	32 (62.7%)	19 (39.6%)	
Back	28 (28.3%)	11 (21.6%)	17 (35.4%)	
Plan to use pillows, stuffed animals, or loose/heavy blankets in sleep environment	23 (23.0%)	14 (27.5%)	9 (18.4%)	χ^2^ = 1.2 (.28)

* p-value for Fisher’s exact test shown; this test is an appropriate alternative to the chi-square test of association with small expected cell counts

### Group comparisons of demographics and safe sleep intentions

The two intervention groups did not differ on maternal age, income, married/partnered status, parity group, high PHQ-2 score status, intention to breastfeed or use of cigarettes, alcohol, or other drugs. The baby box group was more likely than the diaper bag group to indicate a race/ethnicity of Mixed/Mestizo (92% vs. 71%, respectively; *p* = .006).

When indicating prenatal infant sleep intentions most participants suggested the baby would sleep in the mother’s bed (76%), on their side (52%) and without soft items or loose/heavy blankets (77%; as shown in Table 2). There were no differences between the groups on infant sleep intention items during pregnancy, although the diaper bag group was somewhat more likely to intend to have the baby sleep on his/her back compared with the baby box group, prior to the education intervention (*p* = .07 for the group comparison of sleeping position).

### Frequencies of each safer sleep choice by group and over time

The focus of the longitudinal modeling is the comparison of the binary sleep safety indicator (i.e., safer sleep, with a score of 2–3 on the number of safe choices for place, position, and items in sleeping area versus less safe sleep, with a score of 0–1 on the same) between groups and over time. However, we were also interested in the patterns of responses between the groups at each timepoint for each of the components of the total score. The percent who indicated the safer sleep option for each of the component items in the safe sleep scale during pregnancy and at each of the post-birth follow-up surveys are shown in Table 3, with separate estimates for each intervention group. For the sleeping place item there was an increase in those choosing a location other than the mother’s bed for the baby box group between prenatal and 1-month postpartum, which declined by 6 months as mothers tended to have infants sleep in their beds with them when they were older. This pattern is quite different in the diaper bag group, with most infants sleeping in the mother’s bed regardless of the time point (prenatal intention, 1- and 6-months postpartum). The infant position of sleeping on their back was most common in both groups during the post-birth period, having increased almost four-fold in the baby box group and doubled in the diaper bag group after AAP education, compared with prenatal intentions. There, baby box and diaper bag groups were more similar both prenatally and postpartum in the percentages having soft items in the place where the infant slept; both groups reported using pillow, stuffed animals or loose heavy blankets more frequently than they had intended prenatally. Relatively few participants assigned to the baby box group reported using the box as an infant sleeping place (29% at 1 month and 8% at 6 months), but at both postpartum assessments all of the mothers who were using the box were also breastfeeding.

**Table 3:** Number and percent indicating intention or behavior relative to safer sleep choice by item, use of the baby box, and breastfeeding by intervention group (*N* = 100)

Reported Safer choice	Baby Box (*n* = 51)			Diaper Bag (*n* = 49)		
	*Prenatal* (*n* = 51) *n (%)*	*1 month* (*n* = 49) *n (%)*	*6 months* (*n* = 49) *n (%)*	*Prenatal* (*n* = 49) *n (%)*	*1 month* (*n* = 46) *n (%)*	*6 months* (*n* = 43) *n (%)*
*Sleep in bassinet, crib, or other*	14 (27.5%)	20 (42.9%)	11 (22.4%)	10 (20.4%)	4 (8.7%)	3 (7.0%)
*Sleep on back*	11 (21.6%)	40 (81.6%)	41 (83.7%)	17 (34.7%)	35 (76.1%)	33 (76.7%)
*Sleep without pillows, stuffed animals, or loose, heavy blankets*	37 (72.5%)	21 (42.9%)	31 (63.3%)	40 (81.6%)	19 (41.3%)	21 (48.8%)
Reported use of the baby box	–	14 (28.6%) ^a^	4 (8.2%) ^a^	–	–	–
*Breastfeeding*						
*Yes, Exclusively*	51 (100%)	35 (71.4%)	33 (68.8%) ^b^	49 (100%)	31 (67.4%)	26 (60.5%)
*Yes, Supplementing*		14 (28.6%)	10 (20.8%) ^b^		15 (32.6%)	16 (37.2%)
*Not breastfeeding*		0 (0.0%)	5 (10.4%) ^b^		0 (0.0%)	1 (2.3%)
*Using the Box + breastfeeding*		14 (100%) ^a^	4 (100%) ^a^			

^a^ At 1 month and 6 months, 14 and 4 mothers in the baby box group reported using the box for their infants sleeping place (28.6 and 8.2%, respectively); at both timepoints, 100% of those who reported using the box were also breastfeeding

^b^ Although 49 participants in the baby box group completed the 6-month survey, one mother skipped the question about type of feeding at 6 months

### GEE modeling for longitudinal group comparisons

The longitudinal model included the main effects of Time (with three timepoints: the third trimester of pregnancy and 1- and 6-months post-birth) and Group (baby box and diaper bag), as well as their interaction. Controlling for demographic and personal maternal factors, the interaction between Time and Group was significant (Type 3 Score test: χ^2^ = 6.0, *p* = .050). This allowed the evaluation of the pre-planned intervention group contrasts. In particular we wanted to assess whether the two groups differed in likelihood of a higher safety score (i.e., a score of 2 or 3) at each of the three timepoints, and the significance of this interaction term allowed this post-hoc evaluation.

**Fig. 2:**
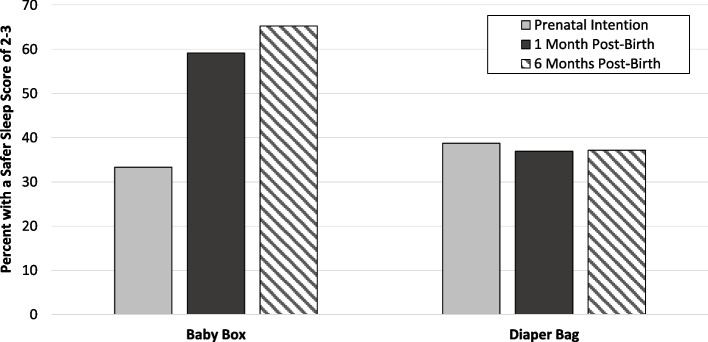
Safe sleep score for each time point

The results of these contrasts suggest that the two groups did not differ in likelihood of a safer sleep score of 2 or 3 at the prenatal intention survey (χ^2^ = 0.7, *p* = .41). However, at the one-month post-birth assessment, there was a group difference in likelihood of a safer sleep score: those in the baby box group were 2.5 times more likely to have a score of 2–3, compared with mothers in the diaper bag group (odds ratio [*OR*] = 2.45 and 95% confidence interval [*CI*]: 1.03–5.86; χ^2^ = 4.1, *p* = .043). The group difference was also present at six-months post-birth: those in the baby box group were 2.9 times more likely to have a score of 2–3, compared with those in the diaper bag group (*OR* = 2.86 and 95% *CI*: 1.16–7.05; χ^2^ = 5.2, *p* = .022). The percent of mothers with a score of at least 2 for each of the timepoints in each group are shown in Fig. 2. The trend of the percentages demonstrates an increase in sleep safety score for the baby box group over time, compared with a relatively flat profile for the diaper bag group.

## Discussion

Prior to this study research regarding safe sleep and the provision of the baby boxes were limited to assessing modes of safe sleep education in addition to provision of the box and for a short window of time or epidemiological data collected as part of a comprehensive maternal/child health initiative. This is the first study investigating the use of the baby boxes to use the same mode of AAP education for all mothers, thus isolating the impact of the provision of the box itself. Results from this study indicated that receiving the box led to safer sleep practices and safer sleep environments. Less than a third (29%) of those who received the box actually reported using it frequently as an overnight sleeping option, however, just having the box in the home may have served as a reminder or cue to practice other safe sleep behaviors, more so than having a diaper bag. Both groups received other reminders such as the safe sleep card, but the box may have served as a larger, more visible reminder for other safe sleep practices based on its purpose and decor. In open-ended feedback many participants remarked that the box was attractive, as they were covered with a baby-friendly decorative film, which may have contributed to positive behavior change as indicated by the Grol’s Framework [17].

Previous researchers have debated whether the box would negatively impact breastfeeding, and our findings suggest they were not impacted, however with such a small sample who used the box and breastfed it is difficult to draw conclusions [23, 28]. Findings related to the uptake and behavior change related to the AAP education were mixed. Both groups had marked increases in sleeping on their backs after the AAP education, however the education regarding soft items in the sleep environment was likely more difficult to operationalize. Open-ended feedback provided some context related to this outcome and provides a direction for future research. Family tradition to keep the infants warm was noted as to why they included items in the sleep environment; they may not have had access to sleep sacks commonly used in the US. Also of note, when asked about why they chose a particular sleep practice, they noted that economic issues, such as not being able to afford a crib or other separate place to sleep, prevented them from taking up the AAP recommendation not to bedshare.

### Strengths and limitations

The primary strength of this study is that it was focused on an understudied and at-risk population, with education and home visits being performed by members of the community. In addition, there is a paucity of research that has evaluated the effect of the provision of a Finnish-style baby box, infant items in conjunction with safe sleep education, compared with education alone. Related to this, the study benefitted from the randomization of participants to the intervention (baby box) and control (diaper bag) groups. As far as limitations this study took place in an under-resourced community, where sleeping space and monetary resources are relatively limited, so these findings are not generalizable to more affluent contexts. There may have been bias introduced through in the community health workers, who were not blinded to the randomization. While 1 month and 6 month AAP safe sleep education was delivered to all participants, the clinical staff may have inadvertently reinforced the safe sleep concepts differentially to participants. Another limitation of the study design is that co-sleeping was considered the least safe practice, but we did not assess the safety of the other sleep surfaces, as mothers reported infants sleeping in many other places in the home, primarily other beds, cribs, bassinets, and hammocks. Additionally, the prenatal intention and post-birth behaviors relative to sleeping practices were self-reported, and this may have been affected by social desirability relative to the education received. The measures used for cigarette and alcohol/other drug use were limited and may have led to an underestimate of these characteristics. Finally, the baby box and diaper bag groups were similar demographically except in ethnicity. Compared with the diaper bag participants the baby box group was more likely to indicate mixed/Mestizo ethnicity, and this may have affected the relationship between intervention group and outcomes. This concern is somewhat mitigated by the inclusion of ethnicity as a control variable in the longitudinal GEE analysis. Larger studies using implementation science frameworks regarding acceptability, feasibility, and cultural resonance of the baby box and other portable sleep devices are warranted. With a larger number of participants in a future study, it would be ideal to also evaluate whether those who report using the baby box for infant sleep differ from those randomized to the same group but who may be using the box for a different purpose, in terms of infant sleep practices. While both of these subgroups may use the box as a visual cue to reinforce safer sleep, the mothers using the box for sleep have a higher ‘dose’ of box exposure and this may affect outcomes.

## Conclusion

While the baby box is not a panacea to the very complex health challenges surrounding the infant sleep environment, they may serve as a prompt or cue to action towards safer sleep practices. Compared to those who were randomized to receive the diaper bag, the provision of the baby box was associated with improved and sustained safer sleep practices (including infant sleep position, sleep location, and loose items or heavy blankets in the sleeping environment) in this small sample in an Ecuadorian community.

In Finland the baby box has a symbolic meaning in that the country invested in the idea of maternal equality and an equal start to life [22, 24]. Perhaps mothers who received the box in Ecuador also felt they were being invested in and part of a larger narrative that they were valued, as was their infants survival, so they may have been more likely to adopt other safer sleep practices to fit into that narrative.

Baskets are very common in Ecuador and this may be a more desirable, culturally resonant, homegrown, and easily reproducible option to have in the family bed while providing some separation, similar to the wahakura basket [18]. However, these baskets may not serve as a reminder of safe sleep if they are perceived as commonplace and not an innovation. In fitting with Grol’s framework, other portable sleep options would need to have an attractive baby-friendly design, be affordable, and be considered an innovation (i.e. different from things used in daily life) to serve as a prompt or nudge towards safe infant sleep practices [17].

Future research should be directed towards testing culturally appropriate safe sleep devices with a larger range of options, but with a focus on those that continue to serve as a prompt to change caregiver behavior and reinforce optimal practices. Further testing and qualitative investigations are warranted. Additionally, future research in safe sleep promotion within Latin America should include follow up education, further exploration of the use of soft items, as well as observations in the home delivered by trusted members of the community; there should also be an emphasis on taking a harm reduction approach, since parents and educations need to work with what they have available in the given context.

## Supplementary Information

**Additional file 1. **Safe Sleep Survey- 5 parts.

## Data Availability

The datasets generated and analyzed during the current study are not publicly available due to the nature and small size of the study but are available from the corresponding author on reasonable request.

## Footnotes

1 Participants were asked to self-identify as Mestizo, White, Afro Ecuadorian, Montubio, or Indigenious
