# Supplementary material for: The provision of the baby box was associated with safe sleep practices in a low-resource community: a randomized control trial in Ecuador
Source: BMC Pediatr. 2023 Jan 19;23:31. doi: 10.1186/s12887-022-03832-y (PMC9850697; doi:10.1186/s12887-022-03832-y)
Supplement: Supplementary file 1 — Additional file 1. Safe Sleep Survey- 5 parts. [file 12887_2022_3832_MOESM1_ESM.pdf]

Safe Sleep Survey- 5 parts

Thank you!

Please fill out the information below.

1 Name of the research personnel entering the data

____

What is number of identification of the participant?

____

Which grouping?

◯ baby box
◯ diaper bag
◯ neither- this the pre-intervention

What survey are you completing?

◯ Pre-Intervention
◯ Post Intervention: Quantitative (1 month)
◯ Home Visit/Observation/Qualitative (1 month)
◯ Post Intervention: Quantitative (6 month)
◯ Home Visit/Observation/Qualitative (6 month)

How old are you?

____

Race/Ethnicity: How do you self- identify?

◯ Mixed race (Mestizo)
◯ White
◯ Indigenious
◯ Montubio
◯ Afro-Ecuadorian (Black)
◯ Other

Other: Race/Ethnicity

____

What week of pregnancy are you?

____

How many pregnancies have you had? (including current pregnancy)

____

How many living children do you have?

____

Did you breastfeed your last baby?

◯ Yes
◯ No

If yes, how long did you breastfeed? (in months)

____

Is there any history of Sudden Infant Death (Crib death) in the family?

◯ Yes
◯ No

If yes, what is your relationship to the child who died ?

____

Do you plan to breastfeed this baby?

◯ Yes
◯ No

Did you plan on becoming pregnant right now? (at the time you became pregnant)

◯ Yes
◯ No
◯ Not sure

Family Questions: On average what is your monthly family income?

◯ Less of equal to 1 minimum wage
◯ More than 1 minimum wage

What can you cover with your monthly family income?

◯ Less than your basic needs
◯ Your basic needs
◯ More than your basic needs

What is the highest grade level of education reached by the father of the baby?

____

What is highest grade level of education you have reached?

____

Are you a single mother (Do not live with the father of the baby)?

◯ Yes
◯ No

How many people live in the home?

____

Who are they?(How are they related to you, not names)

____

Do you smoke?

◯ Yes
◯ No

In the last year, have you ever drunk alcohol or used drugs more than you meant to?

◯ Yes
◯ No

Have you felt you wanted or needed to cut down on your drinking or drug use in the last year?

◯ Yes
◯ No

Over the past two weeks, how often did you have little interest or pleasure in doing things?

◯ Not at all
◯ Several Days
◯ More than half of the days
◯ Nearly Everyday

Over the past two weeks, how often did you feel down, depressed, or hopeless?

◯ Not at all
◯ Several Days
◯ More than half of the days
◯ Nearly every day

Which room is the baby going to sleep overnight?

◯ Mother's room
◯ Other room

In the room, where will your baby sleep?

◯ Mother's bed
◯ Bassinet
◯ Crib
◯ Other

If other, where?

____

Why would you choose this place to put your baby to sleep?

What is the position that the baby should sleep in?

◯ On belly
◯ On side
◯ On back

Why would you choose this position?

Do you plan to let the baby sleep with loose or heavy blankets, pillows, stuffed animals?

◯ Yes
◯ No

If yes, why do you plan to let them sleep with these items?

Sleep Practices- last baby: How often did your last baby sleep in the mother's room overnight?

◯ Always
◯ Very often
◯ Sometimes
◯ Rarely
◯ Never

How often did your last baby sleep in rooms other than with the mother?

◯ Always
◯ Very often
◯ Sometimes
◯ Rarely
◯ Never

In the room, how often did your last baby sleep in bed with the mother?

◯ Always
◯ Very often
◯ Sometimes
◯ Rarely
◯ Never

In the room, how often did your last baby sleep overnight in a bassinet?

◯ Always
◯ Very often
◯ Sometimes
◯ Rarely
◯ Never

In the room, how often did your last baby sleep overnight in a crib?

◯ Always
◯ Very often
◯ Sometimes
◯ Rarely
◯ Never

Other rooms, where and how often?

How often did you put your last baby to sleep on his belly?

◯ Always
◯ Very Often
◯ Sometimes
◯ Rarely
◯ Never

On his side?

◯ Always
◯ Very Often
◯ Sometimes
◯ Rarely
◯ Never

On his back?

◯ Always
◯ Very Often
◯ Sometimes
◯ Rarely
◯ Never

Did you put your last baby to sleep with loose or heavy blankets, pillows or stuffed animals?

◯ Always
◯ Very often
◯ Sometimes
◯ Rarely
◯ Never

Post-Survey: How many weeks of pregnancy were you when you delivered your baby?

____

How many months is your baby?

____

How many prenatal visits did you have? (total pregnancy)

____

How did you deliver the baby?

◯ Vaginal ◯ C-section

Where did you have your baby?

◯ Hospital ◯ Home ◯ Other

Other option for delivery of baby

____

Did the baby stay in the hospital longer than you did?

◯ Yes ◯ No

If yes, why was the baby hospitalized?

____

Does your baby have any health problems

◯ Yes ◯ No

If yes, what is the health problem?

____

Are you breastfeeding your baby?

◯ Yes, exclusively ◯ Yes, supplementing with formula ◯ No

If no, did you ever breastfeed the baby? how long?(in months)

____

Is your child exposed to tobacco smoke?

◯ Always ◯ Very Often ◯ Sometimes ◯ Rarely ◯ Never

Do you smoke?

◯ Yes ◯ No

Over the past two weeks, how often have you had little interest or pleasure in doing things?

◯ Not at all
◯ Several Days
◯ More than half of the days
◯ Nearly every day

Over the past two weeks, how often have you felt down, depressed, or hopeless?

◯ Not at all
◯ Several Days
◯ More than half of the days
◯ Nearly Everyday

Are you currently working? (outside of the home)

◯ Yes ◯ No

If no, are you planning to work outside of the home or go back to school?

◯ Yes ◯ No

If yes, when will you go back to work or school? (months)

____

Does anyone else beside you take care of your baby?

◯ Yes ◯ No

If yes, who helps with the baby, choose all that apply.

☐ Father of the baby
☐ Grandparent of the baby
☐ Babysitter
☐ Aunt or cousin
☐ Other

If other; who takes care of your baby?

____

Nighttime room: How often does your baby sleep overnight in the mother's room?

◯ Always
◯ Very Often
◯ Sometimes
◯ Rarely
◯ Never

Nighttime room: How often does the baby sleep in another room?

◯ Always
◯ Very often
◯ Sometimes
◯ Rarely
◯ Never

In the room, how often does the baby sleep in the mother's bed?

◯ Always
◯ Very often
◯ Sometimes
◯ Rarely
◯ Never

In the room, how often does the baby sleep in a bassinet?

◯ Always
◯ Very often
◯ Sometimes
◯ Rarely
◯ Never

In the room, how often does the baby sleep in the crib?

◯ Always
◯ Very often
◯ Sometimes
◯ Rarely
◯ Never

In the room, how often does the baby sleep in a baby box?

◯ Always
◯ Very often
◯ Sometimes
◯ Rarely
◯ Never

Other: Does the baby sleep in something else another sleep surface? what and how often?

____

Night Position: Does your baby sleep on his on his belly at night and how often?

◯ Always
◯ Very often
◯ Sometimes
◯ Rarely
◯ Never

Night position: Does your baby sleep on his on his side and how often?

◯ Always
◯ Very often
◯ Sometimes
◯ Rarely
◯ Never

Does your baby sleep on his back at night and how often?

◯ Always
◯ Very often
◯ Sometimes
◯ Rarely
◯ Never

Is the baby sleeping with loose or heavy blankets, pillows or stuffed animals?

◯ Always
◯ Very often
◯ Sometimes
◯ Rarely
◯ Never

Day time sleep room: Does the baby sleep in the mother's room?

◯ Always
◯ Very often
◯ Sometimes
◯ Rarely
◯ Never

Day time sleep room: Does the baby sleep in a different/other room than the mothers?

◯ Always
◯ Very often
◯ Sometimes
◯ Rarely
◯ Never

Day: In the room, how often does the baby sleep in the mother's bed?

◯ Always
◯ Very often
◯ Sometimes
◯ Rarely
◯ Never

Day: How often does the baby sleep in a bassinet?

◯ Always
◯ Very often
◯ Sometimes
◯ Rarely
◯ Never

Day: How often does the baby sleep in a crib?

◯ Always
◯ Very often
◯ Sometimes
◯ Rarely
◯ Never

Day: How often does the baby sleep in a baby box?

◯ Always
◯ Very often
◯ Sometimes
◯ Rarely
◯ Never

Day: What and how often does the baby sleep on a different surface?

Day: How often does the baby sleep on his belly?

◯ Always
◯ Very often
◯ Sometimes
◯ Rarely
◯ Never

Day: How often does the baby sleep on his side?

◯ Always
◯ Very often
◯ Sometimes
◯ Rarely
◯ Never

Day: How often does the baby sleep on his back?

◯ Always
◯ Very often
◯ Sometimes
◯ Rarely
◯ Never

How often is the baby sleeping with loose or heavy blankets, stuffed animals, or pillows?

◯ Always
◯ Very often
◯ Sometimes
◯ Rarely
◯ Never

Who do you trust regarding infant care recommendations? (all that apply)

☐ Family
☐ Friends
☐ Health care providers
☐ Other

Other: Who do you trust?

____

What part of the education on safe sleeping practices was most helpful to you? (all that apply)

☐ Video
☐ Handout
☐ Advise by nurse
☐ None

Post-Survey #2: How many weeks of pregnancy were you when you delivered your baby?

____

How many months is your baby?

____

How many prenatal visits did you have? (total pregnancy)

____

How did you deliver the baby?

○ Vaginal ○ C-section

Where did you have your baby?

○ Hospital ○ Home ○ Other

Other option for delivery of baby

____

Did the baby stay in the hospital longer than you did?

○ Yes ○ No

If yes, why was the baby hospitalized?

____

Does your baby have any health problems

○ Yes ○ No

If yes, what is the health problem?

____

Are you breastfeeding your baby?

◯ Yes, exclusively ◯ Yes, supplementing with formula ◯ No

If no, did you ever breastfeed the baby? how long?(in months)

____

Do you smoke?

◯ Yes ◯ No

Is your child exposed to tobacco smoke?

◯ Yes ◯ No

Over the past two weeks, how often have you had little interest or pleasure in doing things?

◯ Not at all
◯ Several Days
◯ More than half of the days
◯ Nearly every day

Over the past two weeks, how often have you felt down, depressed, or hopeless?

◯ Not at all
◯ Several Days
◯ More than half of the days
◯ Nearly Everyday

Are you currently working? (outside of the home)

◯ Yes ◯ No

If no, are you planning to work outside of the home or go back to school?

◯ Yes ◯ No

If yes, when will you go back to work or school? (months)

____

Does anyone else beside you take care of your baby?

◯ Yes ◯ No

If yes, choose all that apply.

☐ Father of the baby
☐ Grandparent of the baby
☐ Babysitter
☐ Aunt or cousin
☐ Other

If other; who takes care of your baby?

____

Nighttime room: How often does your baby sleep overnight in the mother's room?

◯ Always
◯ Very Often
◯ Sometimes
◯ Rarely
◯ Never

Nighttime room: How often does the baby sleep in another room?

◯ Always
◯ Very often
◯ Sometimes
◯ Rarely
◯ Never

In the room, how often does the baby sleep the mother's bed ?

◯ Always
◯ Very often
◯ Sometimes
◯ Rarely
◯ Never

In the room, how often does the baby sleep in a bassinet?

◯ Always
◯ Very often
◯ Sometimes
◯ Rarely
◯ Never

In the room, how often does the baby sleep in the crib?

◯ Always
◯ Very often
◯ Sometimes
◯ Rarely
◯ Never

In the room, how often does the baby sleep in a baby box?

◯ Always
◯ Very often
◯ Sometimes
◯ Rarely
◯ Never

Other: Does the baby sleep in something else another sleep surface? what and how often?

____

Night Position: Does your baby sleep on his on his belly at night and how often?

◯ Always
◯ Very often
◯ Sometimes
◯ Rarely
◯ Never

Night position: Does your baby sleep on his on his side and how often?

◯ Always
◯ Very often
◯ Sometimes
◯ Rarely
◯ Never

Day: How often does the baby sleep on his back?

◯ Always
◯ Very often
◯ Sometimes
◯ Rarely
◯ Never

Is the baby sleeping with loose or heavy blankets, pillows or stuffed animals?

◯ Always
◯ Very often
◯ Sometimes
◯ Rarely
◯ Never

Day time sleep room: Does the baby sleep in the mother's room?

◯ Always
◯ Very often
◯ Sometimes
◯ Rarely
◯ Never

Day time sleep room: Does the baby sleep in a different/other room than the mothers?

◯ Always
◯ Very often
◯ Sometimes
◯ Rarely
◯ Never

Day: In the room, how often does the baby sleep in the mother's bed?

◯ Always
◯ Very often
◯ Sometimes
◯ Rarely
◯ Never

Day: How often does the baby sleep in a bassinet?

◯ Always
◯ Very often
◯ Sometimes
◯ Rarely
◯ Never

Day: How often does the baby sleep in a crib?

◯ Always
◯ Very often
◯ Sometimes
◯ Rarely
◯ Never

Day: How often does the baby sleep in a baby box?

◯ Always
◯ Very often
◯ Sometimes
◯ Rarely
◯ Never

Day: What and how often does the baby sleep on a different surface?

Day: How often does the baby sleep on his belly?

◯ Always
◯ Very often
◯ Sometimes
◯ Rarely
◯ Never

Day: How often does the baby sleep on his side?

◯ Always
◯ Very often
◯ Sometimes
◯ Rarely
◯ Never

Does your baby sleep on his back at night and how often?

◯ Always
◯ Very often
◯ Sometimes
◯ Rarely
◯ Never

How often is the baby sleeping with loose or heavy blankets, stuffed animals, or pillows?

◯ Always
◯ Very often
◯ Sometimes
◯ Rarely
◯ Never

Who do you trust regarding infant care recommendations? (all that apply)

☐ Family
☐ Friends
☐ Health care providers
☐ Other

Other: Who do you trust?

____

What part of the education on safe sleeping practices was most helpful to you? (all that apply)

☐ Video
☐ Handout
☐ Advise by nurse
☐ None

Baby's age in months?

____

Number of children?

____

Mother's age?

____

Does the mother have either of the following?

☐ Baby box
☐ Diaper bag
☐ Neither

If they have the baby box,
What do you like about the baby box?

What do you dislike about the baby box?

Observation: If the participant is using the baby box as sleeping surface, please check the baby box for any signs of damage.

◯ Baby box is in excellent condition. There is not damage. Encourage participant to continue using it until baby outgrows it. Review with participant safety precautions.
◯ Baby box is in good condition. It has some minor damage that does not affect box structure or inside walls. (i.e: minor peeling or scratch on outside wall). Encourage participant to continue using it until baby outgrows it. Review with participant safety precautions.
◯ There is damage to the box structure or peeling of the inside walls. Ask the participant to stop using the baby box as sleeping surface and discuss with her other options to avoid bed-sharing.

If baby box has minor or major damage, please report here. What happened to the box?

____

Qualitative: Where and how does your baby sleep?

Qualitative: Why did you choose this? What are some of the things that influenced your decision to put the baby to sleep this way?

Qualitative: What are some barriers to doing some of the things you learned at the HoH clinic?

Qualitative: If the mother is promoting safe sleep;What are some things that make it easier for you to do some of the things you learned about sleeping at the HaH clinic?

Was the baby awake or asleep for observation during the visit?

◯ awake ◯ asleep

Observation; In which room was the baby sleeping?

◯ Mother's room
◯ Other room

Observation: Where was the baby sleeping?

◯ Mother's bed
◯ Bassinet
◯ Crib
◯ Baby Box
◯ Other

Other: where was the baby sleeping

____

Observation: Which position was the baby sleeping?

◯ Belly
◯ Side
◯ Back

Observation: Does the baby have loose or heavy blankets, pillows or stuffed animals on the surface where the baby was sleeping?

◯ Yes ◯ No

Home Visit #2: Baby's age in months?

____

Number of children?

____

Mother's age

____

Does the mother have either of the following?

◯ Baby box
◯ Diaper bag
◯ Neither

If they have the baby box,
What do you like about the baby box?

What do you dislike about the baby box?

Observation: If the participant is using the baby box as sleeping surface, please check the baby box for any signs of damage.

◯ Baby box is in excellent condition. There is not damage. Encourage participant to continue using it until baby outgrows it. Review with participant safety precautions.
◯ Baby box is in good condition. It has some minor damage that does not affect box structure or inside walls. (i.e: minor peeling or scratch on outside wall). Encourage participant to continue using it until baby outgrows it. Review with participant safety precautions.
◯ There is damage to the box structure or peeling of the inside walls. Ask the participant to stop using the baby box as sleeping surface and discuss with her other options to avoid bed-sharing.

If baby box has minor or major damage, please report here. What happened to the box?

____

Qualitative: Where and how does your baby sleep?

Qualitative: Why did you choose this? What are some of the things that influenced your decision to put the baby to sleep this way?

Qualitative: What are some barriers to doing some of the things you learned at the HoH clinic?

Qualitative: If the mother is promoting safe sleep;What are some things that make it easier for you to do some of the things you learned about sleeping at the HaH clinic?

Was the baby asleep for observation during the visit?

◯ Yes ◯ No

The next 4 questions are things you notice during interview (do not fill out for participant to see)

In which room was the baby sleeping?

◯ Mother's Room
◯ Other Room

Observation: What surface is the mother putting the baby to sleep?

◯ Crib
◯ Bassinet
◯ Baby Box
◯ Mother's bed
◯ Other

Other: what surface? How often?

____

Observation: What position is the mother placing the baby to sleep?

◯ Belly
◯ Side
◯ Back

Observation: Does the baby have loose or heavy blankets, pillows or stuffed animals on the surface where the baby was sleeping?

◯ Yes ◯ No
